# The EH1 motif in metazoan transcription factors

**DOI:** 10.1186/1471-2164-6-169

**Published:** 2005-11-27

**Authors:** Richard R Copley

**Affiliations:** 1 Wellcome Trust Centre for Human Genetics, Roosevelt Drive, Oxford OX3 7BN, UK

## Abstract

**Background:**

The Engrailed Homology 1 (EH1) motif is a small region, believed to have evolved convergently in homeobox and forkhead containing proteins, that interacts with the *Drosophila *protein groucho (*C. elegans *unc-37, Human Transducin-like Enhancers of Split). The small size of the motif makes its reliable identification by computational means difficult. I have systematically searched the predicted proteomes of *Drosophila*, *C. elegans *and human for further instances of the motif.

**Results:**

Using motif identification methods and database searching techniques, I delimit which homeobox and forkhead domain containing proteins also have likely EH1 motifs. I show that despite low database search scores, there is a significant association of the motif with transcription factor function. I further show that likely EH1 motifs are found in combination with T-Box, Zinc Finger and Doublesex domains as well as discussing other plausible candidate associations. I identify strong candidate EH1 motifs in basal metazoan phyla.

**Conclusion:**

Candidate EH1 motifs exist in combination with a variety of transcription factor domains, suggesting that these proteins have repressor functions. The distribution of the EH1 motif is suggestive of convergent evolution, although in many cases, the motif has been conserved throughout bilaterian orthologs. Groucho mediated repression was established prior to the evolution of bilateria.

## Background

The Engrailed Homology 1 (EH1) motif is a short (<10 amino acids) region, initially found in engrailed (en) and other homeobox containing proteins, that mediates transcriptional repression via interaction with the WD40 repeat containing groucho (Gro) [1,2]. Shimeld [3] proposed that the EH1 motif of Smith and Jaynes was shared with various forkhead (FH/HNF-3) containing transcription factors. The short size of the motif, however, suggests that it may occur by chance in many different protein families. Shimeld did not demonstrate statistically significant sequence similarity between the motifs from the homeobox- and forkhead-containing families. However, the human orthologs of groucho (the transducin-like enhancer of split proteins) have been shown to interact with FOXA2 via a region of sequence containing an EH1 motif, clearly demonstrating the biological relevance of the sequence similarity [4].

In this article I search systematically for instances of the EH1 motif in homeobox and forkhead containing genes and go on to demonstrate that the EH1 motif is also found in proteins containing T-box, Doublesex Motif (DM) and Zn finger domains. I show that within metazoan genomes, the observed association of the motif with transcription factor function is statistically significant. The location of the motif in members of the same transcription factor family is often non-homologous, occurring both N- and C-terminal to the DNA binding domain, suggesting that the presence of the motif is, in part, due to convergent evolution, as proposed by Shimeld; the conservation within orthologs points to many of these convergences predating the last common ancestor of the bilateria.

## Results and Discussion

### Significant association of EH1 motif with transcription factor function

**Figure 1:**
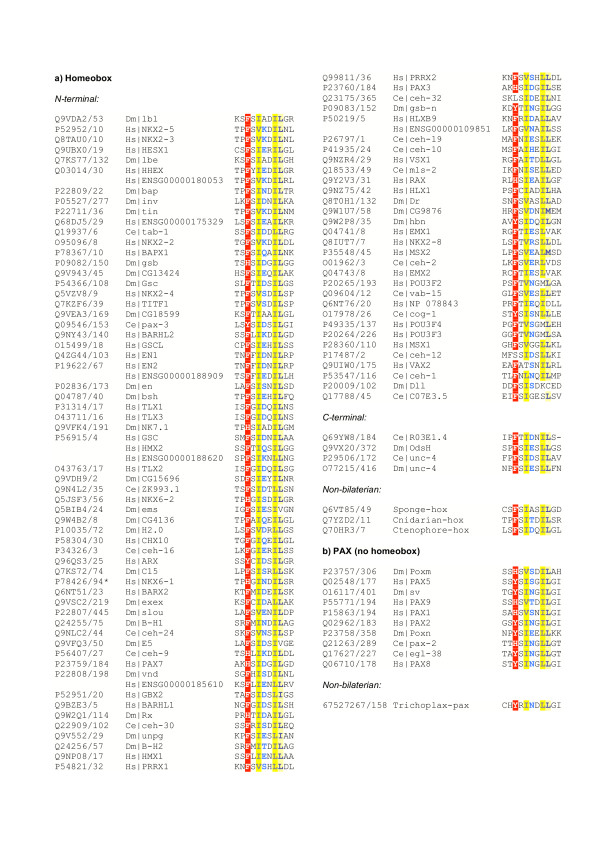
Alignments of putative EH1 motifs in a) Homeobox and b) Paired box domain containing proteins, subdivided by domain partners and orientation, with representative non-bilaterian sequences included. Alignments were derived from meme searches, as described in text. Conserved aromatic residues (FHYW) are coloured white on a red background ('a' in the consensus). Conserved aliphatic residues (ILV), black on a yellow background. ('I' in the consensus) Conserved big residues (EFHIKLMQRWY) blue on a light yellow background ('b' in the consensus). Conservation is calculated over the full alignment of sequences in figures 1 and 2. The figure was produced using the Chroma program [41]. Gene names are standard HUGO Gene Nommenclature Committee, flybase or wormbase symbols where available, otherwise accessions for their respective databases. When available Uniprot protein accessions are also given [42], along with the starting residue of the motif.

**Figure 2:**
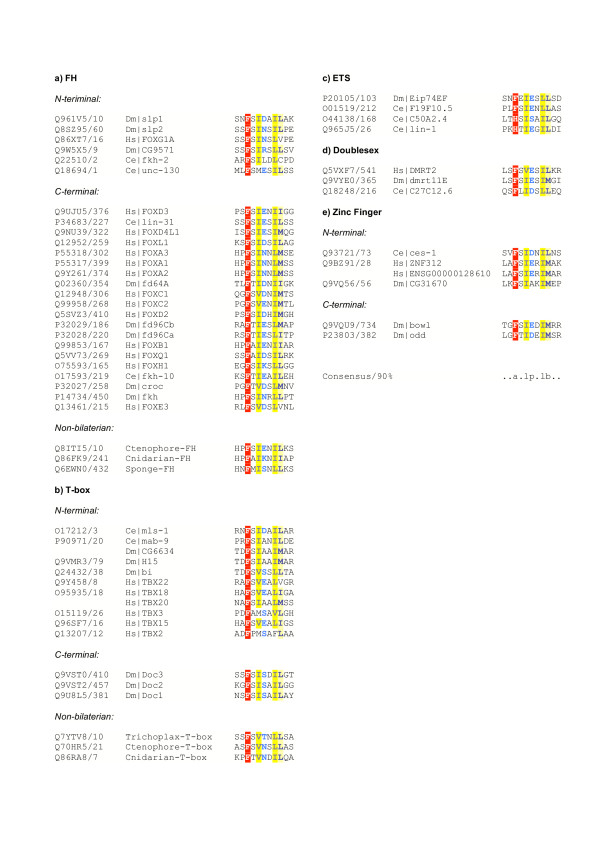
Alignments of putative EH1 motifs in a) Forkhead b) T-box c) ETS d) Doublesex and e) Zinc finger containing proteins. Alignment 'a' was derived from a meme search, as described in text. Sub-alignments b-e were derived from HMMER searches with the EH1^hox ^HMM. Other details as for Figure 1.

I searched for sequence motifs in homeobox containing transcription factors taken from the proteins of human, *Drosophila melanogaster *and *Caenorhabditis elegans*, by first masking known Pfam domains [5], and then using the expectation maximization algorithm implemented in the meme program [6]. The first non-subfamily specific motif identified corresponded to previously known examples and new instances of, the EH1 motif (see Figure 1a), in 100 sites, with an E-value of < 10^-126^. I then applied the same approach to Forkhead containing transcription factors, identifying 25 sites with a combined E-value of < 10^-31 ^(Figure 2a). These motifs also appeared to conform to the consensus of the EH1 motif, as previously reported by Shimeld [3].

**Figure 3:**
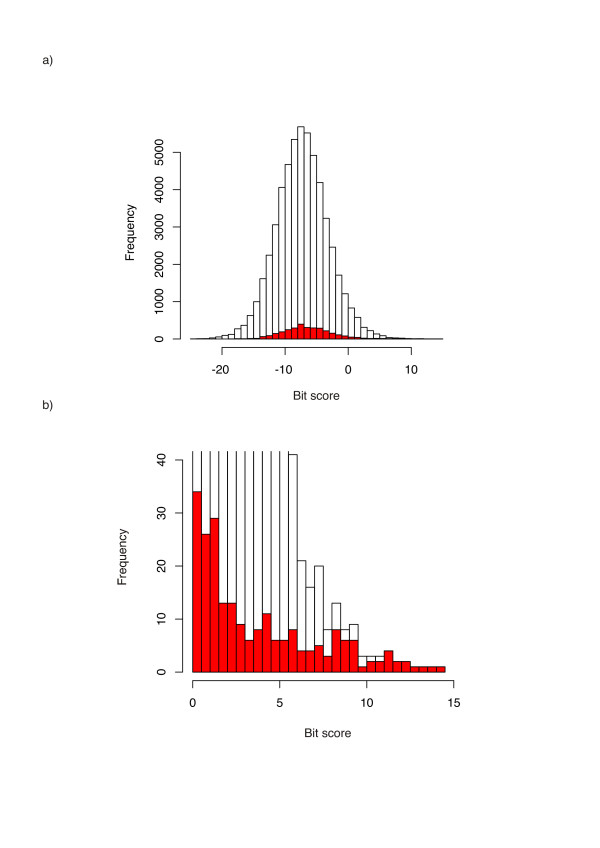
a) Distribution of HMMER bit scores for the database search of EH1^hox ^HMM against the combined proteomes of human, *D. melanogaster *and *C. elegans*. Counts from scores from transcription factors (see methods) have been coloured red – i.e. the proportion of a bar coloured red is equal to the proportion of transcription factors. Scores from proteins containing a homeobox domain (interpro accession IPR001356), from which the EH1^hox ^HMM was derived, have been excluded, b) as for 'a', but rescaled to show region of biological relevance. High scoring hits are greatly enriched in specific transcription factor families. For scores ≥ 5.0 bits, there are 68 transcription factors and 142 non-transcription factors; for scores <5.0 bits, 3075 transcription factors and 51513 non-transcription factors giving a chi-square test p-value statistic of P < 0.0001 – the statistical significance is discussed more fully in the text.

To further investigate the significance of this similarity, I constructed hidden Markov models (HMM) of the motif (EH1^hox ^& EH1^fh^) which I then searched against the complete set of predicted proteins from human, *D. melanogaster *&*C. elegans*. The highest scoring non homeobox containing domain match of EH1^hox ^was a Forkhead protein (human FOXL1), and the second highest scoring non-Forkhead containing match of EH1^fh ^was to a homeobox containing protein (*D*. *melanogaster *inv). In both cases, nearly all the high scoring hits were to proteins containing domains with transcription factor function (see Figure 3). Among the best scoring matches of the EH1^hox ^searches were several T-box (TBOX), Doublesex Motif (DM), Zinc finger (ZnF_C2H2) and ETS containing proteins (domain names as per SMART, Figure 2b–e) [7,8]. Excluding hits to homeobox containing proteins, but otherwise including all scores, the overall significance of the association of transcription factor function with higher scores to the EH1^hox ^HMM is P < 10^-47^, using a logistic regression model which tests association between score and transcription factor annotation (see methods and supplementary file 1 for raw data). The association remains significant when scores derived from Forkhead and PAX domain containing proteins are also excluded (P < 10^-34^). This indicates that, although the scores associated with any individual EH1-like motif may not be statistically significant, overall, we would not see so many EH1-like sequences co-occurring with DNA binding domains if their co-occurrence were governed simply by chance – there is, therefore, likely to be a functional reason for these partnerships. In the following sections, I review the higher scoring associations detected here in the light of known gene functions.

### EH1 motifs in homeobox and forkhead containing proteins

The presence of EH1 motifs within various homeobox, and to a lesser extent, forkhead containing proteins has been widely reported, although not systematically studied [3]. I found EH1-like motifs co-occurring with 3 major groupings of homeobox sub-types: the extended-hox class, typified by *Drosophila *engrailed (en); the paired class, including *Drosophila *goosecoid (gsc), and the NK class, including *Drosophila *tinman (tin) [1,9,10] (see [11] for a description of these broad classes). Related to the paired class homeobox domains, a number of genes containing PAIRED domains only (i.e. the PAX domain of SMART [7]) were also found to contain EH1-like motifs (see Figure 1b). With only a few exceptions, outlined below, the EH1-like motif occurs N-terminal to the homeobox domain and C-terminal to the PAIRED domain when present. A number of these proteins have been shown to interact with groucho or its orthologs e.g. *C. elegans *cog-1 [12], vertebrate Nkx proteins [13], *Drosophila *engrailed (en) and goosecoid (gsc) [2,14], and in high throughput assays *Drosophila *invected (inv) and and ladybird late (Ibl) [15].

A handful of EH1-like motifs are found C-terminal to homeobox domains. Of these, the best characterized is *C. elegans *unc-4, which has been shown to interact with the groucho ortholog unc-37 [16]; the *Drosophila *ortholog unc-4 also interacts with groucho in high throughput experiments [15]. The C-terminal EH1-like motif is conserved in the closely related *Drosophila *paralog OdsH. The gene prediction for the human ortholog of unc-4 (ensembl gene identifier ENSG00000164853) appears to be artefactually truncated, but the mouse ortholog (Uncx4.1 ENSMUSG00000029546) and corrected human gene models, contain EH1-like motifs both N & C-terminal to the homeobox domain. Taken together with the fact that in the majority of related homeobox containing proteins the EH1-like motifs are N-terminal, this suggests that the N-terminal motif has been lost in *Drosophila *and *C. elegans *unc-4 orthologs.

EH1-like motifs also occur N- and C-terminal to Forkhead domains. The N-terminal class consists of the sloppy-paired genes (slp1 and slp2) of *Drosophila *and orthologous or closely related sequences: human FOXG1, and *Drosophila *CG9571; the *C. elegans *ortholog fkh-2 contains an EH1-like motif although a cysteine residue causes a low score. The C-terminal class consists of an apparent clade including the human FOXA, FOXB, FOXC and FOXD genes (Figure 2a), although if the EH1 motif was present in the common ancestor of this clade, multiple losses must have later occurred (see [17] for a Forkhead domain phylogeny). The situation is complicated somewhat by an EH1-like motif at the N-terminus of *C. elegans *unc-130 i.e. in the FOXD like family. The EH1 motif in slp1 has been shown to interact with groucho [18], and FOXA type genes have been shown to interact with human groucho orthologs [4].

### EH1 motifs in novel domain contexts

Assuming a conservative per-domain cutoff score of 10.0 bits for true matches to the EH1^hox ^model (see Figure 3), yields hits to proteins containing T-box domains (highest score 13.1 bits); Doublesex (DM) domains (highest score 11.6 bits) and C2H2 Zinc fingers (highest score 11.2 bits). Also of note was a further match at 9.4 bits, to an ETS domain containing protein. Prompted by these similarities I further investigated the presence of EH1-like motifs in these families, looking for high scoring matches to the EH1^hox ^HMM that were conserved in closely related genes.

#### T-box containing proteins

I identified likely EH1 motifs co-occurring with T-Box domains in two distinct contexts (Figure 2b). The motif occurs C-terminal to the T-box in the *Drosophila *dorsocross proteins Doc1, Doc2 and Doc3. It is found N-terminal to the T-box in 11 proteins including mls-1 and mab-9 from *C. elegans; *H15, mid/nmr2 and bi/omd from *Drosophila*; in humans there are strong matches to TBX18, TBX20 and TBX22 and more marginal matches to TBX3 and TBX2. Although, to the best of my knowledge, none of these proteins has been shown to interact with groucho or its orthologs, several are known to act as transcriptional repressors: for instance, in murine heart development, Tbx20 represses Tbx2 which in turn represses Nmyc [19,20]; the Dorsocross genes from *Drosophila *repress wingless and ladybird [21], and Doc itself is repressed by mid/nmr2 [22]. The human proteins TBX1 and TBX10, and *Drosophila *org-1 which are closely related to those above, do not appear to contain EH1 motifs. The human T (brachyury) protein contains a motif broadly similar to the EH1 consensus: LQ**Y**R**V**DH**LL**SA in a comparable N-terminal location to those found in other T-box containing proteins. Although this motif scores poorly against EH1^hox ^(-0.1 bits), the homologous regions from other T orthologs (for instance, the non-bilaterian sequences discussed below) provide a more persuasive case for the presence of a functioning EH1 motif in these proteins.

#### Zinc finger containing proteins

The highest scoring match of EH1^hox ^to a C2H2 zinc finger containing protein, was ces-1 from *C. elegans *(bit score 11.2); this protein interacts with the groucho ortholog unc-37 [[23], #54] and can act as a repressor [24]. The putative EH1 motif is at the N-terminal end of ces-1. In contrast, the *Drosophila *proteins bowl and odd have EH1-like motifs at their C-terminal ends (with bit scores of 10.9 & 8.4 respectively). In neither case is there direct evidence from high throughput studies of an interaction with groucho, but both can function as repressors [25]. The human protein ZNF312 (bit score 8.6) is the ortholog of zebrafish Fezl, which contains an EH1 motif essential for repressor activity [26] – this motif is conserved in the human paralog ENSG00000128610 and likely *Drosophila *ortholog CG31670 (bit scores of 8.4 & 5.1) (Figure 2e).

#### Doublesex motif containing proteins

The Doublesex Motif (DM) was first found in proteins controlling sexual differentiation in *Drosophila*. Two DM containing proteins were confidently predicted to contain EH1-like motifs – human DMRT2 (bit score 11.6), and *Drosophila *dmrt11e (bit score 11.2) – these are likely orthologs; a *C. elegans *protein, C27C12.6 contained a weaker match (bit score 6.6) (Figure 2d). The molecular function of these proteins is unknown.

#### Other potential associations with transcription factor domains

Although scoring less highly than some non-transcription factor hits, another intriguing association is with the ETS domain. The three uncharatcerized *C. elegans *paralogs F19F10.5, F19F10.1 & C50A2.4 contain C-terminal matches to the EH1 motifs (bit scores 9.4, 2.3 & 7.4), and two other ETS proteins, *C. elegans *lin-1, and *Drosophila *Eip74EF, both have relatively high scoring matches (bit scores 6.5 & 6.6) (Figure 2c). A high scoring protein that is not annotated as a transcription factor (as it contains no interpro domains) is *Drosophila *Hairless (H) with a score of 8.3 bits. Experimental work has previously confirmed the presence of an EH1-like motif (SS**Y**S**I**HS**LL**GG) within H that is responsible for its interaction with groucho [27]. The *Drosophila *protein Dorsal has been reported to interact with groucho via an EH1-like motif [28] – this region (NG**P**T**L**SN**LL**SF) is markedly different to those reported here, having a low score against EH1^hox ^(-10.7 bits) and so may better be regarded as a, so far, unique type of groucho interaction motif.

### Evolutionary considerations

#### Convergent evolution

The EH1 motif is found N- and C-terminal to homeobox, forkhead, T-box and Zn finger protein domains. Clearly, as the locations of the EH1 motif are non-homologous, the N- and C-terminal associations must have occurred independently. The short size of the motif makes it tempting to speculate that the motif itself may have arisen independently (i.e. in repeated cases it may have evolved within sequence that was already part of the gene, rather than via a recombination event). The strongest evidence for this is that, in general, the majority of domain combinations occur in a fixed N to C orientation, suggesting that recombination events combining domains are relatively rare [29,30]. The fact that we would here have many such events suggests that the alternative hypothesis of independent invention is more appropriate.

#### Pre-bilaterian origins of association with different transcription factors

*Groucho *is orthologous to the *C. elegans *unc-37 gene, and the four human paralogs TLE1-4 (Transducin Like Enhancer of split). An ortholog is also found in the cnidarian *Hydra mangipapillata *(e.g. the EST with gi 47137860, data not shown), and certain cnidarian homeobox containing genes also contain an EH1-like motif, suggesting groucho/EH1 mediated repression pre-dates the split between diplobasts and triplobasts; indeed, a sponge Bar/Bsh like homeobox containing protein (i.e. protein gi: 33641772) [31] also contains an EH1-like motif, as does paxb from the non-bilaterian placozoan *Trichoplax adhaerens *[32] and a Tlx-like protein from a ctenophore (gi: 38602653), suggesting the repression system was in place in the earliest animals (see [33] for a discussion of early metazoan evolution). I find high scoring EH1-like motifs in Forkhead domain containing proteins from sponges, cnidarians and ctenophores, in both the C-terminal (FOXA-D clade) (region II in [34]) and N-terminal (FOXG, sloppy paired clade) varieties (reported as 'HPFSI' in [35]). The presumed ortholog of 'T' from the *Trichoplax adhaerens *[36] contains an EH1-like motif (8.6 bits). These results suggest that groucho mediated repression using a variety of transcription factors was widespread in the last common ancestor of the metazoa.

## Conclusion

Candidate EH1 motifs exist in combination with a variety of transcription factor domains, suggesting that these proteins have roles as repressors of transcriptional activity. The distribution of the EH1 motif is suggestive of a number of instances of convergent evolution, although in many cases the motif has been conserved throughout bilaterian orthologs. Together with the existence of a cnidarian Groucho ortholog, this leads to the conclusion that EH1/Groucho mediated repression was established prior to the evolution of bilateria.

## Methods

Proteomes were derived from ensembl 32 (human NCBI 35, *C. elegans *wormbase 140, *Drosophila *BDGP 4) [37]. In cases of multiple splice variants, the one with the most exons was included (or the longest in the case of ties). Transcription factor activity was taken as the presence of the gene ontology accession GO:0003700 associated with an interpro domain predicted for the protein [38]. These data were also taken from ensembl. Although C2H2 subtype Zn fingers are not annotated by Interpro as transcription factors they are DNA binding and frequently have this role, so have been included in the transcription factor set. Bit scores reported in the text are for comparisons of the EH1^hox ^HMM against the target sequence using the HMMER software package [39].

The association of transcription factor function (coded as a dichotomous variable, *t*, taking the values 1 [transcription factor] or 0 [non-transcription factor]) with the bit score, *x*, of the EH1^hox ^HMM, was tested using a logistic regression model implemented in the glm() function of the R package [40]). I fitted the model

Prob(*t *= 1) = exp(*a *+ *bx*)/(1 + exp(*a *+ *bx*))

The coefficients *a*, *b *were estimated from the data by maximum-likelihood. The hypothesis of no association is equivalent to testing if *b = *0.

Where inferences of orthology are made, they are based on clear-cut separation of BLAST scores or alignment-based phylogenies.

## Authors' contributions

RRC performed the analysis and wrote the paper.

## Supplementary Material

Additional File 1 Each non-homeobox containing protein in the database is classified as either being a transcription factor or not (see methods), and its score against the EH1^hox ^HMM is given. Click here for file
